# Consistent mutational paths predict eukaryotic thermostability

**DOI:** 10.1186/1471-2148-13-7

**Published:** 2013-01-10

**Authors:** Vera van Noort, Bettina Bradatsch, Manimozhiyan Arumugam, Stefan Amlacher, Gert Bange, Chris Creevey, Sebastian Falk, Daniel R Mende, Irmgard Sinning, Ed Hurt, Peer Bork

**Affiliations:** 1 European Molecular Biology Laboratory, Meyerhofstrasse 1, Heidelberg, 69117, Germany; 2 Biochemie-Zentrum der Universität Heidelberg, Im Neuenheimer Feld 328, Heidelberg, D-69120, Germany; 3 Teagasc Animal Bioscience Centre, Grange, Dunsany, Co. Meath, Ireland; 4 Max-Delbrück-Centre for Molecular Medicine, Berlin-Buch, Germany; 5 Current address: LOEWE Zentrum für synthetische Mikrobiologie, Phillips-University-Marburg, Marburg, Germany

**Keywords:** Thermophily, Comparative genomics, Protein engineering, Eukaryotes, Fungi

## Abstract

**Background:**

Proteomes of thermophilic prokaryotes have been instrumental in structural biology and successfully exploited in biotechnology, however many proteins required for eukaryotic cell function are absent from bacteria or archaea. With *Chaetomium thermophilum, Thielavia terrestris* and *Thielavia heterothallica* three genome sequences of thermophilic eukaryotes have been published.

**Results:**

Studying the genomes and proteomes of these thermophilic fungi, we found common strategies of thermal adaptation across the different kingdoms of Life, including amino acid biases and a reduced genome size. A phylogenetics-guided comparison of thermophilic proteomes with those of other, mesophilic Sordariomycetes revealed consistent amino acid substitutions associated to thermophily that were also present in an independent lineage of thermophilic fungi. The most consistent pattern is the substitution of lysine by arginine, which we could find in almost all lineages but has not been extensively used in protein stability engineering. By exploiting mutational paths towards the thermophiles, we could predict particular amino acid residues in individual proteins that contribute to thermostability and validated some of them experimentally. By determining the three-dimensional structure of an exemplar protein from *C. thermophilum* (Arx1), we could also characterise the molecular consequences of some of these mutations.

**Conclusions:**

The comparative analysis of these three genomes not only enhances our understanding of the evolution of thermophily, but also provides new ways to engineer protein stability.

## Background

Proteins from thermophilic organisms are not only stable at higher temperatures, but are also generally more stable than their mesophilic counterparts. Therefore they are scientifically valuable, e.g. for biochemical and structural studies, and have multiple applications in industry [1]. However, many proteins exclusively occur in eukaryotes, and only a few of the latter are thermophilic (defined as having an optimal growth temperature [OGT] above 50°C; [2]. Recently, the first eukaryotic thermophilic genome, *Chaetomium thermophilum,* gave first insights into the potential for structural biology [3]. Now with two more genomes, *Thielavia terrestris* and *Thielavia heterothallica*[4] being published, comparative analysis of their thermophilic nature can be performed.

*Thielavia terrestris* and *Thielavia heterothallica* (anamorph *Myceliophtora thermophila*) are filamentous fungi of the class Sordariomycetes [4] which can be found in ‘unnatural’ habitats like compost. Their natural habitat seems to be in soils such as in semi-arid grasslands in New Mexico [5]. They are common in multiple microhabitats in this region, where high summer temperatures in combination with episodes of substantial precipitation provide favourable conditions [5]. *Chaetomium thermophilum* is a widely distributed soil-inhabiting fungus and a thermophile in accordance with its lifestyle in self-heating composting plant material [6]. It can also be found in composting urban solid waste [7,8] and wood-chip piles [9,10]. *C. thermophilum* is a member of the large genus of *Chaetomium*, also within the Sordariomycetes, that are found in soil, air, and plant debris [11]. Close relatives of these thermophilic fungi are the mesophilic mould fungus *Chaetomium globosum* (OGT 24°C), a frequent indoor contaminant that produces mycotoxins and acts as an allergen [11], and *Neurospora crassa*, another mesophilic filamentous fungus of which the genome has been published [12].

Due to their thermostable nature, proteins from thermophilic fungi have recently gained considerable attention in industry and structural biology. Several crystal structures of proteins from these thermophilic fungi have been determined such as those of two beta 1,4-galactanases from *T.heterothallica*[13], a glycoside hydrolase from *T. terrestris*[14], and Get3, Get4 and beta 1,4-xylanase from *C. thermophilum*[15-17]. The paper-industry utilizes members of the beta 1,4-xylanase family for bio-bleaching of kraft-pulp [18,19]. The biotechnological potential of *C. thermophilum* is also illustrated by the purification and characterization of its thermostable superoxide dismutase (SOD) [20], an enzyme which is utilized in cosmetic products to reduce free radical damage to the skin. Furthermore, the genomes of *C. thermophilum, T. terrestris* and *T. heterothallica* provide a source of thermostable cellulolytic enzymes, such as the glycoside hydrolases that can be used in the production of third-generation biofuels [14].

Here, we identify commonalities and differences of thermophilic adaptation between eukaryotes and prokaryotes and exploit the close relationship of the thermophilic to mesophilic fungi to gain detailed insight into the molecular evolution of thermophily. By comparing the genomes of thermophilic fungi to each other and to mesophilic relatives we can clarify the evolutionary trajectory that has been obscured by inconsistent naming conventions [4] and determine whether there are independent events of gain of thermophily in these fungi. We further use the observed adaptation biases to predict mutations that can increase the thermostability of proteins and verify them experimentally.

## Results and discussion

### Taxonomic position of thermophilic fungi within Chaetomiaceae

**Figure 1:**
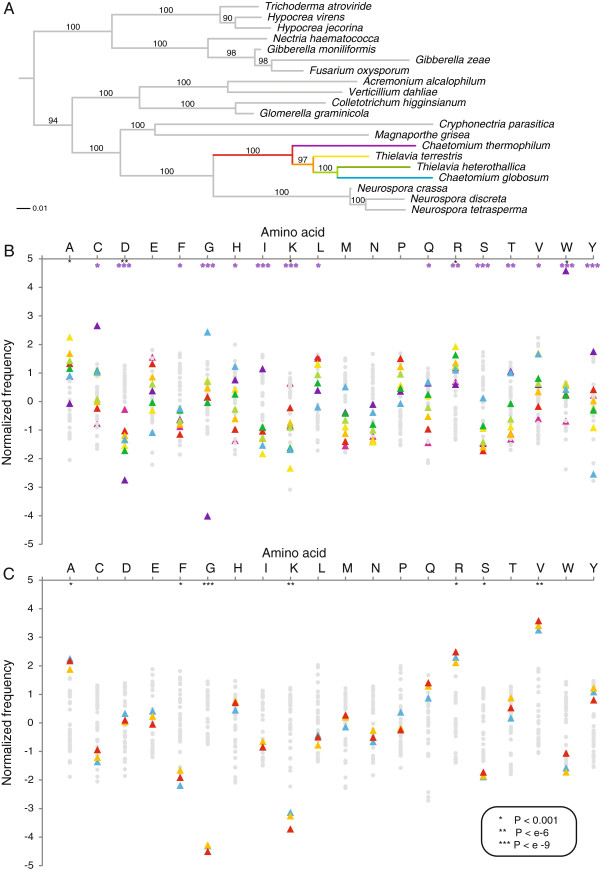
**Evolution of thermophily in Sordariomycetes.A)** Maximum likelihood tree of Sordariomycetes based on 40 marker genes. Numbers on branches indicate bootstrap values. The mesophilic fungus *Chaetomium globosum* clusters within the thermophiles with 100% bootstrap support. **B)** Amino acid frequencies in single copy proteins and reconstructed ancestral proteins were normalized for average and standard deviation. Coloured triangles are according to the colours of the branches in A: blue Cgl; dark green Tht; light green ancestor of Cgl and Tht; yellow Tht; orange ancestor of Tte and Cgl; purple Cth; red ancestor of Cth and Cgl. Black stars indicate significant differences between the thermophiles and the mesohpiles, purple stars between *C. thermophilum* and the mesophiles **C)** Amino acid frequencies of single copy proteins within the sub Class of Eurotiomycetidae, containing two sequenced thermophiles; *Thermomyces lanuginosis* and *Talaromyces thermophilus*. Black stars indicate significant differences between the thermophiles and the mesohpiles.

To determine the phylogenetic relationships between thermophilic and mesophilic fungi of the Sordariomycetes, we searched for the presence of 40 phylogenetic marker genes [21] in published and unpublished genomes of this clade using Hidden Markov Models (HMMs; see Materials and Methods), and used bootstrapping and Maximum Likelihood to calculate a phylogenetic tree (Figure 1A). Despite the different naming, the three thermophilic species closely group together, implying that the most parsimonious scenario is a single invention of thermophily. However, *Chaetomium globosum*, the closest mesophilic neighbour of these three thermophilic species is monophyletic within the thermophiles with 97% bootstrap support and most likely lost thermophily. As this was surprising, we also generated phylogenetic trees using 2,064 universal single copy orthologs established specifically for the Sordariomycetes using the eggNOG pipeline [22]. We indeed could confirm the taxonomic positions implying loss of thermophily (Additional file 1: Figure S1).Thus, by studying this lineage we can gain insight both in the gain and loss of thermophily.

### Genome reduction in thermophiles

The genomes of *C. thermophilum* (Cth), *T. terrestris* (Tte) and *T. heterothallica* (Tht) are significantly smaller than their close mesophilic relatives such as *Chaetomium globosum* (Cgl) and *Neurospora crassa* (Ncr). In agreement with previous studies of prokaryotic thermophiles, the genome size reduction is due mainly to fewer protein coding genes (Cth 7,267; Tte 9,813; Tht 9,110 vs Cgl 11,124 and Ncr 10,620), but also to shorter introns and shorter intergenic regions (Additional file 1: Figure S2) and [4]. Since *C. globosum* is derived from the ancestor of these three species, there are two possibilities. Either, this ancestor had a small genome and *C. globosum* has gained genes by duplications or horizontal transfers or the three thermophiles have independently lost genes in a parallel adaptation process. Although larger genomes in the outgroups makes a loss and gain scenario more likely, we investigated all orthologous groups from the complete genomes of 20 members of the Sordariomycetes (sorNOGs) to clarify the gene content evolution of eukaryotic thermophiles. Firstly, we analysed the phylogenetic presence/absence patterns of these sorNOGs. In total, 4,542 protein coding genes are present in equal copy numbers in each of the four species Cth, Tte, Tht and Cgl. Present in one copy but absent from either of the four are 330 (Cgl), 125 (Tte), 130 (Tht) and 440 (Cth) orthologs, meaning that lineage specific loss alone does not account for the differences in genome size. *C. globosum* specific duplications are responsible for ca. 150 extra genes. It must be noted that some lineage specific losses may also be accounted for by difference in genome quality, but the tendencies will remain.

On the other hand, there are 845 orthologous groups covering 1,004 genes of *C. globosum* that are absent in all three others. These numbers are 181 (190), 325 (353) and 543 (579) orthologous groups (genes) for Cth, Tte and Tht. The difference in genome size can thus partly be assigned to these orthologous groups. A large number of these are related to transposable elements, including 30 transposases, 74 reverse transcriptases, 30 DNA helicases. The lack of these elements in the thermophilic fungi may indicate that transposition is unfavourable at higher temperatures.

Oxygenases and enzymes hydrolyzing complex sugars are in particular frequently lost in the thermophiles. This does not always mean that metabolic capabilities are completely absent; often multigene families in *N. crassa* and *C. globosum* have only one counterpart in *C. thermophilum*, but also non-homologous isoforms are reduced to one enzyme, implying a reduction in robustness. Proteins that are completely missing in *C. thermophilum* but not in the two *Thielavias* include WC1, WC2 and FRQ which are involved in the regulation of the circadian clock [23,24]. We hypothesize that due to the localization far inside the compost away from light (implied by the high temperature optimum) the day-night rhythm does not play a role for *C. thermophilum*.

There are no major gene family expansions in the thermophiles compared to their relatives, only a few orthologous groups have been slightly expanded against the reductionist trend. The majority of them are uncharacterized, but some indicate life style adaptation such as a cellobiose dehydrogenase of which *C. thermophilum* has three copies and *C. globosum* and *N. crassa* only two, reflecting an increased wood degradation capacity. *T. terrestris* has five copies of a S-adenosyl-L-methionine (SAM) dependent methyltransferase that is likely to employ arsenite as substrate where its relatives have only one or two. The largest lineage specific expansion in *T. heterothallica* is an orthologous group with three copies of a scytalone dehydratase involved in fungal melanin biosynthesis. Melanin provides resistance to UV radiation, drought and high temperatures [25] and thus this expansion likely represents a thermophilic adaptation. The lack of major expansions suggests that the metabolisms of the thermophilic fungi have not undergone major niche adaptations requiring additional functionality, and that the dominating adaptation was indeed the one to higher temperatures.

### Convergent evolution of thermophily across all domains of life

It has been previously shown that the amino acid frequencies vary with the OGT, specifically the summed frequency of the amino acids IVYWREL shows the highest correlation with OGT in both bacteria and archaea [26]. In these domains of life, the ancestor was likely a thermophile and adaptation happened to colder environments [21].

We therefore investigated whether the molecular principles of thermostability in fungal proteins are similar. In alignments of the 2,064 single copy orthologs universal in Sordariomycetes (see Methods and Table 1 for species list), we find that the total frequency of IVYWREL amino acids as in thermophilic archaea and bacteria is significantly higher in *C. thermophilum* compared to the other Sordariomycetes but not in *T. heterothallica* and *T. terrestris* (P-value < E^-16^). This is explained mainly by the extremely high frequencies of isoleucines, tryptophans and tyrosines in *C. thermophilum* (Figure 1B). Addition of these large hydrophobic amino acids is likely to play a role in filling the hydrophobic cores of proteins (e.g. [27] and below). Only part of this signal, the increased levels of arginine and tryptophane are present in all three thermophiles. Specific to the two *Thielavias* is an enrichment in alanine. Furthermore, consistent differences between the three thermophilic and the mesophilic fungi are lower frequencies of aspartic acids and lysines in the thermophiles (Figure 1B). The more extreme reduction of genome size together with the IVYWREL bias in *C. thermophilum* leads us to hypothesize that this fungus might survive at higher temperatures than the two *Thielavias* for which optimal growth temperatures have not been published yet.

**Table 1:** Genomes used for amino acid bias analysis

**Sordariomycetes**	**Eurotiomycetidae**
*Acremonium alcalophilum*	*Arthroderma otae*
*Chaetomium globosum*	*Aspergillus aculeatus*
*Chaetomium thermophilum*	*Aspergillus carbonarius*
*Colletotrichum higginsianum*	*Aspergillus fumigatus*
*Cryphonectria parasitica*	*Aspergillus niger*
*Fusarium oxysporum*	*Aspergillus terreus*
*Gibberella moniliformis*	*Blastomyces dermatitidis*
*Gibberella zeae*	*Coccidioides immitis h538*
*Glomerella graminicola*	*Coccidioides immitis rs*
*Hypocrea jecorina*	*Emericella nidulans*
*Hypocrea virens*	*Histoplasma capsulatum h143*
*Magnaporthe grisea*	*Histoplasma capsulatum h88*
*Nectria haematococca*	*Microsporum gypseum*
*Neurospora crassa*	*Paracoccidioides brasiliensis*
*Neurospora discreta*	*Talaromyces thermophilus*
*Neurospora tetrasperma*	*Thermomyces lanuginosus*
*Thielavia heterothallica*	*Trichophyton equinum*
*Thielavia terrestris*	*Trichophyton rubrum*
*Trichoderma atroviride*	*Trichophyton tonsurans*
*Verticillium dahliae*	*Trichophyton verrucosum*
	*Uncinocarpus reesii*

Analyzing the amino acid frequencies from bacterial (Additional file 1: Table S1) and archaeal (Additional file 1: Table S2) clades with thermophilic members, we observe a striking difference with eukaryotes; an overrepresentation of cysteines in *C. thermophilum* proteins (Figure 1B); in total 15% of cysteines in aligned positions are unique to *C. thermophilum*. The major categorized roles of cysteines are in catalytic residues, disulfide bridges and metal binding (e.g. zinc fingers), whereby the latter two contribute to folding and stability. Cysteines have also been shown to contribute to thermal stability in their free form, when they form interactions inside the core of a protein [28]. This unique adaptation of *C. thermophilum* may be another indication that its proteins are better adapted to high temperatures than the other two thermophilic Sordariomycetes. Another difference between prokaryotes and eukaryotes that we observe is that glycines are strongly depleted in *C. thermophilum* whereas they are enriched in *C. globosum* compared to the complete clade of Sordariomycetes. The exchange of alanines with glycines has been shown to destabilize alpha-helices, particularly in the center of the helix [29]. It seems as if *C. globosum* has indeed used this strategy to make proteins less thermo-stable, and *C. thermophilum* has evolved in the opposite direction, lowering its glycine content.

We verified the generalizability of these trends by examining two more unpublished thermophilic fungal genomes, *Thermomyces lanuginosus* and *Talaromyces thermophilus* of the subclass Eurotiomycetidae, a different fungal clade that also includes *Aspergillus fumigatus* and *Emericella nidulans.* Compared to their mesophilic neighbours, these species both have a significantly higher total frequency of IVYWREL amino acids (P < 1e-7). They also show a depletion of glycines and significant enrichment in arginines and alanines (Figure 1C) consistent with the biases in the thermophilic Sordariomycetes. This shows that some of the trends are indeed universal between different clades of fungi.

### Mutational paths towards thermophily

**Figure 2:**
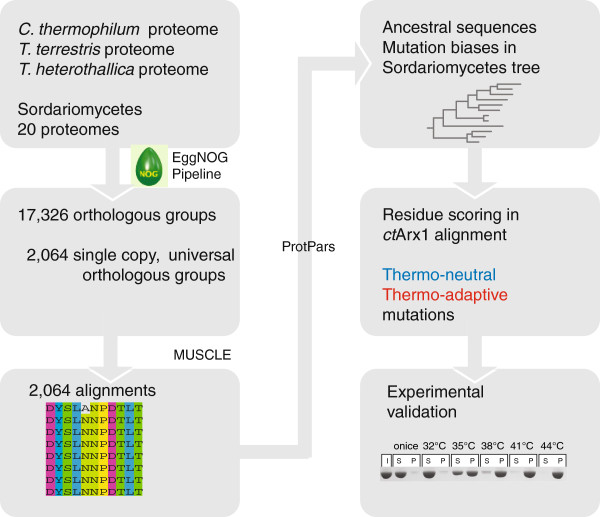
Workflow for finding biases and a scoring scheme of thermophilic mutations.

**Figure 3:**
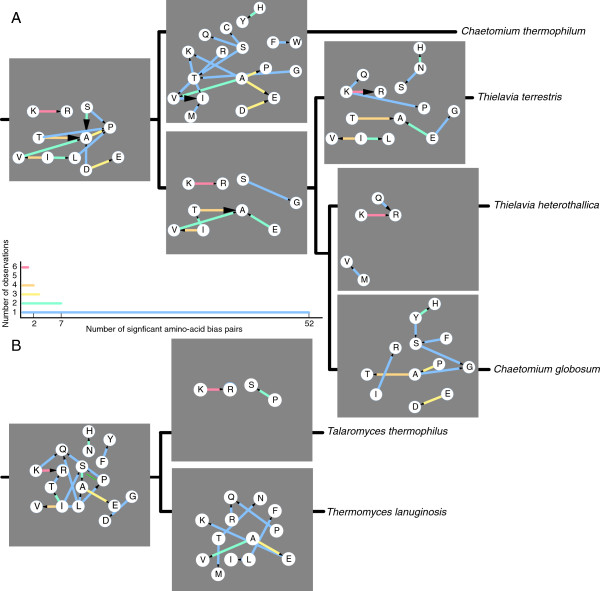
**Biases in pairwise mutation rates.** Alignments were made of all single copy orthologous groups and subjected to parsimony reconstruction using the marker gene tree as a guide tree. The frequencies of mutations between all pairs of amino acids were analysed. The ratios between all pairs of amino acids were compared to the ratios in the reconstructed phylogeny between mesophiles. In principle, it is expected that there are as many mutations from X to Y as from Y to X. Thus a bionomial test can be used to assess a bias. However, there are also biases in the complete groups of Sordariomycetes and Eurotiomycetidae. Therefore the expected ratio is not set to 1:1, but to the actual ratio in the mesophilic neighboring species. Amino acid pairs with significant bias are connected by coloured lines, with an arrowhead proportional to the bias in the direction of the more frequent mutations. A histogram of bias pairs is showing how often among all nine branches the bias pair is observed, colours are used to connect the amino acids in **A)** thermophilic Sordariomycetes and **B)** thermophilic Eurotiomycetidae.

In contrast to thermophilic prokaryotes, the genomes of thermophilic fungi have very close, known mesophilic relatives and thus, for the first time, we can trace and quantify the mutational paths by which the differences in amino acid composition arise (Methods). We therefore have quantified the mutation biases between pairs of amino acids in all branches of the Sordariomycetes tree and determined how different they are in one branch compared to the rest of the tree (Figure 2). This is similar to but more specific than a previous analysis on biases between pairs of prokaryotic thermophiles and mesophiles [30]. In the prokaryote study the mesophile-thermophile species pairs were much more dissimilar than our mesophile-thermophile relatives and thus there would be a large effect of multiple substitutions at each site resulting in 139 out of 190 amino acid pairs showing a bias. Likely because of the difference in evolutionary distance we observe a smaller number of significantly biased amino acid pairs (65 out of 190) in the branches leading to thermophilic fungi (Figure 3). We observed that mutation bias between several small amino acids and prolines has led to higher frequency of prolines already in the ancestor of the thermophilic Sordariomycetes (Figure 3A). Analyzing the amino acid frequencies from bacterial (Additional file 1: Table S1) and archaeal (Additional file 1: Table S2) clades with thermophilic members we also found that proline frequency is increasing with higher OGT (Additional file 1: Table S3) which is significant in bacteria but not in archaea; Prolines make the protein structure more rigid and less likely to unfold as has been shown before in case studies [31-33]. This strengthens the hypothesis that the ancestor of the thermophilic Sordariomycetes and *C. globosum* was also thermophilic. Furthermore, there are significantly more mutations from lysine to arginine than vice versa; the replacement of lysine by argnine has been shown to lead to less fluctuations in side groups [34]. This lysine to arginine bias is present in four out of five branches leading to thermophily in Sordariomycetes (Figure 3A) [30]. Other consistent biases are between aspartic and glutamic acid as well as between threonine and alanine, where we observe the opposite trend in the branch where the thermophily is lost, leading to *C. globosum*. The increased level of lysine to arginine mutations as hallmark of eukaryotic thermophilic adaptation was confirmed in two out of three branches in Eurotiomycetidae leading to the two monophyletic thermophilic species *T. lanuginosus* and *T. thermophilus***(**Figure 3B). Moreover the strong bias of serine to alanine is also present in these species. Apart from these consistent biases, there are also unique, individual biases in the branches. As in prokaryotes it seems to be also the case in eukaryotes that increased thermostability can be achieved in many ways depending on the context.

Considering the consistent biases, we analysed particular residues in orthologous groups shared between Sordariomycetes and Eurotiomycetidae. We found that where the same biases exist, the overlap between positions where e.g. arginines have been introduced instead of lysines is significant but small, i.e. 92 out of a total of 7,335 positions that are changed from lysine to arginine in any of the thermophiles are shared between all five. This leads us to believe there are some positions that are more likely to increase stability and may even be essential; however mutations at many other positions can contribute independently.

In contrast to prokaryotes where GC content has been found to cause a bias in amino acid frequencies of lysines and arginines [35,36], as previously reported in these fungi the GC content does not differ significantly between mesophiles and thermophiles [3]. There is an elevated GC content at the third codon position as reported by [4], however the frequencies of G at C and the third codon position do not differ between lysine and arginine. Therefore, in thermophilic fungi the lysine-arginine bias has arisen independently of the GC content.

### Scoring scheme for adaptive mutations

Based on our observations, we developed a scoring scheme to give weight to individual mutations for their contribution to thermophily (Figure 2). We used the mutation bias between pairs of amino acids in the branches leading to the thermophilic ancestor as well as to *C. thermophilum* to arrive at these scores (see Methods). We predict that those positions with a high score are responsible for the thermophilic adaptation of individual proteins. In this way, we can distinguish which thermophile specific mutations are likely to be adaptive and which are likely to be neutral. Since the thermophilic nature of proteins has been lost in *C. globosum*, we can also predict which mutations have been responsible for this loss. In this way we predicted 38,385 thermophilic adaptive mutations in 2,064 single copy proteins for which we could trace the ancestral amino acid sequences.

### Mutations important for thermophilic stability

**Figure 4:**
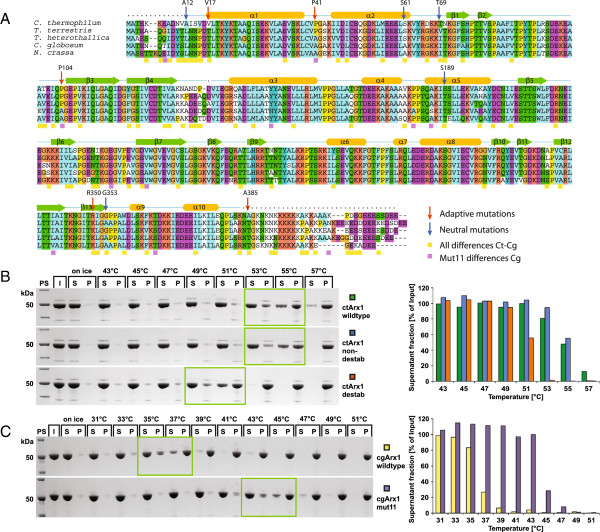
**Amino acids responsible for thermostability**. **A)** Alignment of Arx1 protein of the three thermophilic Sordariomycetes, *C. globosum* and *N. crassa*. Arrows represent positions of introduced mutations that we predict to be adaptive to thermophily (red) or neutral (blue). Secondary structure elements are indicated above the alignment (cylinder: α-helix; arrow: β–strand; line: loop regions; dotted line: not solved in crystal structure). Violet squares represent mutations in the *cg*Arx1mut11 and yellow squares represent all other differences between ctArx1 and cgArx1. Amino acids are colored with the default color scheme of ClustalX [38]. **B)** Arx1 from *C. thermophilum* (*ct*Arx1) is thermostable and its thermostability can be influenced by mutating residues predicted to be adaptive for thermophily. Recombinant wildtype *ct*Arx1 and mutant *ct*Arx1-nondestab and *ct*Arx1-destab proteins were affinity-purified and incubated at the indicated temperatures for one hour. Then the proteins were separated into supernatant (S) and pellet (P) fractions by centrifugation and subjected to SDS-PAGE and Coomassie stain in comparison to the input (I). PS, protein standard. The five mutations present in *ct*Arx1-nondestab and *ct*Arx1-destab were predicted to be neutral or adaptive mutations to thermophily. The fraction of protein present in the supernatant fraction was analyzed by quantifying the Coomassie stained protein bands with Aida Image Analyzer v. 4.00. The input was set to 100%. **C)** Recombinant cgArx1mut11 protein contains mutations in 11 residues predicted to have destabilized the protein of C. globosum. Thermostability analysis of recombinant cgArx1 and cgArgx1mut11 as described in (B).

**Figure 5:**
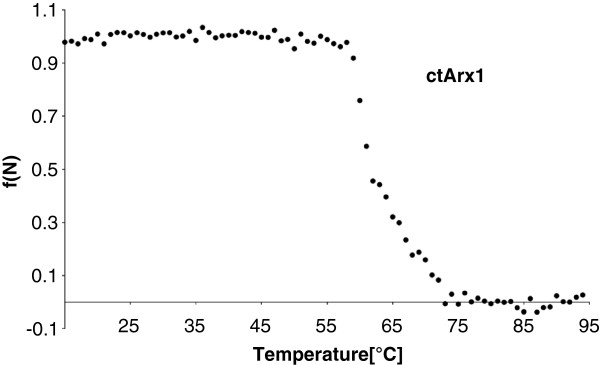
**CD spectrum of *ct*Arx1.** The protein core of *ct*Arx1 unfolds around 62°C. Unfolding of *ct*Arx1 at ~0.1 mg/ml concentration is monitored by CD at 222.6 nm under a temperature gradient. The normalized ellipticity is plotted against the temperature.

To validate some of these predictions experimentally, we applied them to a protein from *C. thermophilum*, which is homologous to yeast pre-ribosomal export factor Arx1 (Associated with Ribosomal eXport complex) [37]. *C. thermophilum* Arx1 (*ct*Arx1) is thermostable (soluble) up to 53°C at a concentration of 8 mg/ml, whereas the Arx1 from the mesophilic *C. globosum* (*cg*Arx1; Figure 4A, B) precipitates already at 35°C (Figure 4C), corresponding to the OGTs of both organisms. Circular dichroism (CD) spectra showed that *ct*Arx1 began to unfold in vitro at 55°C and reached complete unfolding at ~70°C (Figure 5). To test whether the predicted adaptive and neutral mutations have an effect on the thermostability of our model protein, we generated two mutant *ct*Arx1 proteins with either five predicted adaptive or five predicted neutral positions in *ct*Arx1 changed to the respective ancestral residues (Figure 4A, B). The predicted non-destabilizing (neutral residues) *ct*Arx1 mutant behaved like wild-type *ct*Arx1 and remained soluble up to 53°C (at 8 mg/ml) and up to 55°C (6-fold diluted; Figure 4B). However, the predicted destabilizing (adaptive residues) *ct*Arx1 mutant remained soluble only up to 49°C (8 mg/ml) and 50°C (6-fold diluted; Figure 4B; Additional file 1: Figure S3). This confirms our prediction scheme to find mutations that increase thermostability. Furthermore, we identified eleven *C. globosum* specific mutations that we think are likely to have destabilized this protein (Figure 4A, C). Introducing the ancestral amino acid for all these eleven mutations indeed increased the temperature at which the protein remained soluble. Thus we could turn back time and create a thermostable from an unstable protein.

### Structural context for adaptive mutations

**Figure 6:**
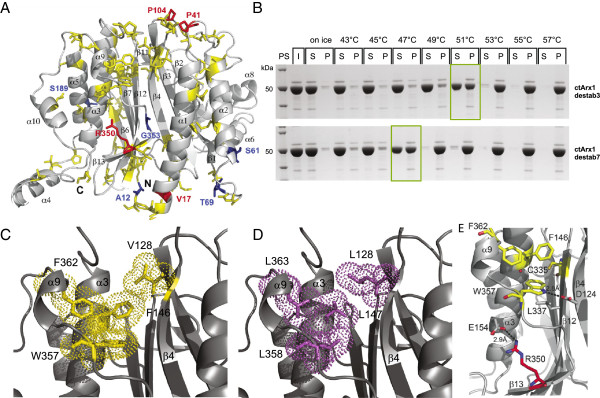
**Hydrophobic core packing in the Arx1 protein. A)** Ribbon representation of the *ct*Arx1 structure. *ct*Arx1 crystallized readily, whereas cgArx1 did not. Adaptive (destabilizing) and neutral (non-destabilizing) amino acids are highlighted in red and blue, respectively. The positions of amino acids, which differ between the cg and *ct*Arx1 proteins, are marked in yellow. The positions of the *ct*Arx1 specific cysteines, C249 and C335, and the prolines, P135 and P182, are indicated by stars and triangles respectively. The N- and C-termini of *ct*Arx1 are indicated by ‘N’ and ‘C’. **B)** Mutation of a hydrophobic patch leads to reduced thermostability of *ct*Arx1. Two *ct*Arx1 mutant proteins containing mutations within the hydrophobic patch described in (C,D,E) are tested for their thermostability in comparison to wild-type *ct*Arx1 at the indicated temperatures and at a protein concentration of 8 mg/ml. *ct*Arx1-destab7 contains the five destabilizing mutations of *ct*Arx1-destab (Figure 4B) and two mutated hydrophobic residues (F146L, W357L), *ct*Arx1-destab3 contains F146L, W357L, and the destabilizing mutation R350K. **C)** A closed hydrophobic stretch of exchanged amino acids in *ct*Arx1 increases the contact between secondary structure elements and close a surface groove in *ct*Arx1. Val128, Phe146, Trp357 and Phe362 of *ct*Arx1 are highlighted in yellow. **D)** The corresponding modeled amino acid residues of cgArx1, Leu128, Leu147, Leu358 and Leu363, are highlighted in magenta. In both panels the volumes of the residues are indicated with small dots. **E)** The adaption of *ct*Arx1 to thermophily is achieved in part by the increase of hydrophobic density and polar/electrostatic contacts with the core of the protein’s scaffold. The adaptive amino acid Arg350 (Lys) is highlighted in red. Hydrophobic animo acids, which differ between the cg and *ct*Arx1 proteins, are marked in yellow. Amino acid side chain interactions are indicated by black, dashed lines.

To reveal mechanistic roles for adaptive residues, we determined the 3D structure of *ct*Arx1 that shares the pita-bread fold with methionine-aminopeptidases [39] and Ebp1 [40] (Figure 6). Expression, purification, crystallization and x-ray structure determination of this protein was successful, supporting the value of *C. thermophilum* as a model system for structural studies. The two selected adaptive proline mutations (P41, P104) indeed occur in loops of *ct*Arx1 (Figure 6A) preventing unfolding as mentioned above [31-33]. Another fundamental concept in thermo-adaptation of proteins is an increased bulkiness of hydrophobic amino acids within the protein core. According to some models, unfolding is due to the transfer of water into the protein hydrophobic core that progressively breaks hydrophobic contacts and swells the protein interior [27]. Direct sequence comparison of *ct*Arx1 with *cg*Arx1 indeed shows that 80% (16/20) of the hydrophobic amino acid exchanges lead to increased bulkiness. Several of these adaptive bulky hydrophobic residues in *ct*Arx1 (F146, F362 and W357) together with V128 form an extended hydrophobic cluster, which together with adaptive C335 leads to a tight packing of helices α3 and α9 to the central beta-sheet (Figure 6A, E). Acquired electrostatic interactions are found between the imino-group of W357 and D124 (β4) linking β4 to α3 more tightly (Figure 6C) and between adaptive R350 in β13 and E154 stabilizing β13 with respect to helix α3. Mutation of F146, W357 and R350 reduces the thermostability of *ct*Arx1 by about 2°C to 51°C (Figure 6B, C). In addition, mutation of the two hydrophobic residues (F146, W357) on top of the five adaptive mutations leads to a further decrease of *ct*Arx1 thermostability to 47°C (Figure 6B, D). Taken together, these examples of adaptive mutations in the context of the 3D structure of *ct*Arx1 illustrate how individual residues and their interactions contribute to a thermophilic adaptation.

## Conclusions

Here, we show that the principles of thermophilic adaptations in fungi are similar to that in prokaryotes, with the notable exception of cysteines that are enriched in *C. thermophilum* and that might contribute to thermophily in several ways. The close relation of mesophilic species allows predicting particular mutations that are directly responsible for thermo-adaptation, which we could confirm experimentally by protein engineering. By solving the 3D structure of a single thermophilic protein (Arx1 of Cth), we could identify three different types of adaptive mutations : (i) loop rigidity by increased proline frequency, (ii) increased protein core hydrophobicity, and (iii) increased electrostatic interactions stabilizing neighboring secondary structure elements.

By now, several structures have been determined already based on *C. thermophilum, T. terrestris* and *T. heterothallica* proteins [13-17] and we and others have determined the thermostable nature of several other proteins [3,41]. This, together with our finding of thousands of mutations towards thermophily in this lineage, implies that the thermostability of proteins is a major contributor to the increased OGT of these organisms, in particular in *C. thermophilum*. *C. thermophilum* is, as are *T. terrestris* and *T. heterothallica* promising resources for (thermo)stable proteins for industrial purposes as well as for biochemical and structural studies that rely on stable eukaryotic proteins and the assembly of complex molecular machines. With experimental tools such as genetic transformation protocols and a number of independent lineages containing thermophilic eukaryotes, a rapidly increased understanding should lead to precise predictions which particular mutation increases thermophily via which mechanisms for a vast amount of important eukaryotic proteins.

## Methods

### Fungal orthologous groups

Published genomes were downloaded from NCBI. Unpublished genomes were downloaded from ftp-sites of the Joint Genome Institute, the BROAD institute and Genome Canada. Non-supervised orthologous groups (NOGs) were constructed for 20 Sordariomycetes and 21 Eurotiomycetidae (Table 1) through identification of reciprocal best BLAST [42] matches and triangular linkage clustering as implemented in eggNOG v2 [22]. This resulted for Sordariomycetes in 17,325 and for Eurotiomycetidae in 14,979 non-supervised orthologous groups (NOGs). Out of 7,227 *C. thermophilum* proteins, we find orthologs in other Sordariomycetes for 7,045 of them. We found 2,064 NOGs that contain exactly one copy from each Sordariomycetes proteome (universal single copy orthologs) and 1,436 that contain exactly one copy from each of the Eurotiomycetidae. HMMs of 40 marker genes [21] were used to search all 20 Sordariomycetes proteomes. These were aligned using MUSCLE [43], pruned with GBLOCKS [44] and a tree was built using RAxML [45]. Trees were displayed using iTOL [46]. The same procedure was applied for all 2,064 universal single copy orthologs.

### Amino acid overrepresentation

Alignments of universal single copy orthologs were made using MUSCLE [43] with standard settings. Amino acid frequencies were counted in all aligned positions that do not contain gaps. The frequencies in *C. thermophilum* were compared against frequencies in mesophilic Sordariomycetes and Z-tests were done to obtain significant differences in all aligned positions. Similarly, T-tests were done to obtain significant differences between the three thermophiles and their ancestral nodes on the one hand and the mesophiles and their ancestral nodes on the other hand. Significant differences are shown as stars in Figure 1B and C. A similar analysis was done in a group of seven bacteria (Additional file 1: Table S1) and in a group of seven archaea (Additional file 1: Table S2) with large variance in the optimal growth temperatures. In this case there was not one species that was thermophilic, therefore, rather than a Z-test, correlations of the AA-frequencies with OGT were calculated (Additional file 1: Table S3) and t-tests were done between the AA-frequencies of (hyper)thermophilic and mesophilic species to obtain significances (Additional file 1: Table S3).

### Mutational paths

Parsimonious reconstructions of ancestral states were made using PROTPARS from the Phylip package [47] with the fungi tree as user tree for all single copy NOG alignments. From the output file, steps at each position were parsed and counted only if they were unambiguous. The frequencies of mutations between all pairs of amino acids were analysed. The ratios between all pairs of amino acids were compared to the ratios in the whole reconstructed phylogeny. In principle, it is expected that there are as many mutations from X to Y as from Y to X. Thus a bionomial test can be used to assess a bias. However, there are also biases in the complete groups of Sordariomycetes and Eurotiomycetidae. Therefore the expected ratio is not set to 1:1, but to the actual ratio in the mesophilic neighboring species.

### Scoring of amino acid substitutions

We developed a scoring scheme to give a weight to individual mutations for their contribution to thermophily. We used the mutation bias between pairs of amino acids to arrive at these scores. We calculate the binomial probability of the number of mutations from amino acid X to Y vs Y to X, given the average ratio between X to Y and Y to X in the whole Sordariomycetes tree. The logarithm of this probability is multiplied by −1 to come to a score S for pair X and Y. If in a phylogenetic reconstruction, there is a mutation from X to Y and there is a significant bias from X to Y, this mutation will get the positive score S, if there is a significant bias from Y to X, it will get the negative score S, otherwise the mutation is not scored.

### Purification of recombinant protein

ORFs for cg*ARX1*, ct*ARX1*, ct*arx1-destab* and ct*arx1-nondestab* were synthesized and sequenced by Eurofins MWG Operon (Ebersberg, Germany) or GenScript (Piscataway, NJ, USA) and subcloned into pET-24a(+) vector. Proteins were expressed in *E. coli* BL21 (DE3) grown in LB-medium at 37°C under vigorous shaking. Cell pellets were resuspended in buffer A (20 mM Hepes-NaOH pH 8.0, 350 mM NaCl, 10 mM KCl, 10 mM MgCl_2_, 40 mM imidazol). Cells were lysed by a Microfluidizer (M-110 L, Microfluidics) and the lysate was cleared by ultracentrifugation at 91,000 × g for 20 minutes. Recombinant protein was purified by Ni-ion affinity chromatography (Ni-NTA-HisTrap, GE-Healthcare) via an N-terminal hexa-histidine tag and eluted with buffer A supplemented with 460 mM imidazol. *ct*Arx1 was further purified by size exclusion chromatography (S200-26/60, GE-Healthcare) in a buffer containing 20 mM Hepes-NaOH pH 8.0, 200 mM NaCl, 10 mM KCl and 10 mM MgCl_2_.

### Crystallization and structure determination of *ct*Arx1

Crystals of *ct*Arx1 were grown at 18°C by the sitting drop vapour diffusion method. Sitting drops were prepared by mixing 0.5 μl of fresh *ct*Arx1 (15 mg/ml) with 0.5 μl of reservoir solution containing 0.2 M LiAcetate and 2.2 M (NH_4_)_2_SO_4_. Prior X-ray analysis crystals were flash-frozen in liquid nitrogen after cryo-protection by transfer into a cryosolution containing mother liquor and 25% v/v glycerol. Data-collection was performed at ID23/1 at the European Synchrotron Radiation Facility in Grenoble (France). Data were processed in iMosflm and Scala [48]. The structure of *ct*Arx1 was solved by molecular replacement using ccp4 implemented PHASER [49] and the crystal structure of Ebp1 as the search model [40]. The structure was manually built in Coot [50] and refined with Refmac5 [51]. Data and refinement statistics are given in Table 2. Figures were generated with Pymol (http://www.pymol.org).

**Table 2:** **Crystal data of Arx1 from *C. thermophilum***

**Data collection**	
Space group	*P*2_1_2_1_2
Unit cell parameters (Å)	192.0, 193.3, 70.9
(°)	90, 90, 90
Resolution (Å)	86.4 – 2.3 (2.42 – 2.3)
R_Merge_^a^	0.127 (0.53)
Unique reflections	118103 (17031)
Completeness (%)	100 (100)
Multiplicity	5.9 (5.9)
<I/σI>	13.7 (4.0)
**Refinement**	
Number of used reflections	112170
Resolution limits (Å)	57.0 -2.3
R factor ^b^ (%)	20.2
Free R factor ^c^ (%)	24.2
Rmsd bond lengths (Å)	0.016
Rmsd bond angles (°)	1.535

**a.** R_merge_ = Σ_h_Σ_j_|I_hj_ - <I_h_>|/Σ_h_Σ_j_, where I_hj_ is the intensity of the jth observation of the unique reflection h.

**b.** R factor = Σ_h_||F_oh_| - |F_ch_||/Σ_h_|F_oh_|, where F_oh_ and F_ch_ are the observed and calculated structure factor amplitudes for reflection h.

**c.** The free R factor is equivalent to the R factor, but is calculated using 5% of reflections excluded from the maximum-likelihood refinement stages.

### Thermostability tests

Thermostabilites of *ct*Arx1 and *cg*Arx1 were determined by testing an *in vitro* aggregation. For this assay, recombinant *ct*Arx1 and *cg*Arx1 were purified from *E. coli* and incubated at the indicated temperatures (see Figure 3B, D, Additional file 1: Figure S7B) for one hour in buffer 2 (50 mM Tris–HCl pH 7.5, 200 mM NaCl, 10 mM KCl, 10 mM MgCl_2_, 5% (v/v) glycerol, 0.01% (v/v) MTG). Following centrifugation at 20,000 rpm at 4°C for 30 minutes, an equivalent sample of the supernatant and the pellet fraction was separated by SDS-polyacrylamide gel electrophoresis (PAGE; NuPAGE 4–12% Bis-Tris Gel, Invitrogen). Proteins were visualized with Coomassie (Brilliant Blue G – colloidal Concentrate, Sigma-Aldrich).

### Circular dichroism

For measuring unfolding of *ct*Arx1 the circular dichroism (CD) was recorded at different temperatures. Dichroism spectra from *ct*Arx1 were recorded at a protein concentration of ~0.1 mg/ml on a Jasco J-810 spectropolarimeter in a 0.1 cm path length cuvette at 20°C. Proteins were exchanged into 10 mM potassium phosphate, pH 7.5. Four scans were measured from 250 to 200 nm in 1 nm increments with a 1 s averaging time and a bandwidth of 1 nm. The scans were averaged, and the buffer spectrum was subtracted. Mean residue ellipticity Θ_MRW_ was calculated according to Equation 1, where Θ is the raw signal in millidegrees, l is path length in cm, n is the number of amino acids, and c is the concentration of the protein in moles per liter.

(1) $$\Theta_{\mathrm{MRW}}=\frac{\Theta }{10\times 1\times n\times c}$$

### Thermal denaturation

Thermal unfolding transitions of *ct*Arx1 were followed by circular dichroism at 222.6 nm with 1 nm bandwidth in 2 mm cells and a heating rate of 1°C per minute using a Jasco J-810 spectropolarimeter in 10 mM potassium phosphate, pH 7.5, at a protein concentration of ~0.1 mg/ml.

## Competing interests

The authors declared that they have no competing interests.

## Authors’ contributions

VvN, MA, CC and DRM performed the computational analyses. BB, SA, GB and SF performed the experiments. PB and EH conceived the study. PB, EH and IS directed the work. VvN, BB and SA wrote the manuscript; all authors were involved in the revision and have read and approved the final manuscript.

## Supplementary Material

Additional file 1 **Table S1.** Bacterial genomes and Optimal Growth Temperature. **Table S2.** Archaeal genomes and Optimal Growth Temperature. **Table S3.** correlations with OGT in bacterial and archaeal clades containing thermophiles. **Figure S1.** Phylogenetic tree of Sordariomycetes. A maximum likelihood tree was calculated with RaXML based on the concatenated alignments of 2,064 single copy orthologs in Sordariomycetes. Numbers on the branches indicate bootstrap support. **Figure S2.** Intergenic length distribution of *N. crassa*, *C.globosum* and *C.thermophilum.* Intergenic regions of *C. thermophilum* (blue) are significantly smaller than *Neurospora crassa* (red) and *Chaetomium globosum* (green), due to genome compaction. **Figure S3.** Thermostability of Wild-type and Mutant *ct*Arx1. The critical temperature for thermostability is higher at lower protein concentration. The thermostability test (in vitro aggregation assay) with ctArx1 mutant proteins was performed at a 6-fold lower concentration (~1.3 mg/ml) than in **Figure **4B*ct*Arx1-nondestabilizing and ctArx1-destabilizing with five neutral or adaptive mutations, respectively (see **Figure **4A, B), and ctArx1 wild-type recombinant proteins were affinity-purified and incubated at the indicated temperatures for 1 hour, separated into supernatant (S) and pellet (P) fractions by centrifugation and subjected to SDS-PAGE and Coomassie stain in comparison to the input (I). PS: protein standard. Click here for file
