# Supplementary material for: Consistent mutational paths predict eukaryotic thermostability
Source: BMC Evol Biol. 2013 Jan 10;13:7. doi: 10.1186/1471-2148-13-7 (PMC3546890; doi:10.1186/1471-2148-13-7)
Supplement: Additional file 1 — Table S1. Bacterial genomes and Optimal Growth Temperature. Table S2. Archaeal genomes and Optimal Growth Temperature. Table S3. correlations with OGT in bacterial and archaeal clades containing thermophiles. Figure S1. Phylogenetic tree of Sordariomycetes. A maximum likelihood tree was calculated with RaXML based on the concatenated alignments of 2,064 single copy orthologs in Sordariomycetes. Numbers on the branches indicate bootstrap support. Figure S2. Intergenic length distribution of N. crassa, C.globosum and C.thermophilum. Intergenic regions of C. thermophilum (blue) are significantly smaller than Neurospora crassa (red) and Chaetomium globosum (green), due to genome compaction. Figure S3. Thermostability of Wild-type and Mutant ctArx1. The critical temperature for thermostability is higher at lower protein concentration. The thermostability test (in vitro aggregation assay) with ctArx1 mutant proteins was performed at a 6-fold lower concentration (~1.3 mg/ml) than in Figure 4B ctArx1-nondestabilizing and ctArx1-destabilizing with five neutral or adaptive mutations, respectively (see Figure 4A, B ), and ctArx1 wild-type recombinant proteins were affinity-purified and incubated at the indicated temperatures for 1 hour, separated into supernatant (S) and pellet (P) fractions by centrifugation and subjected to SDS-PAGE and Coomassie stain in comparison to the input (I). PS: protein standard. [file 1471-2148-13-7-S1.docx]

**Consistent mutational paths predict eukaryotic thermostability**

# Supplementary Tables

Table S1 Bacterial genomes and Optimal Growth Temperature

| **Species** | **OGT** |
| --- | --- |
| *Carboxydothermus hydrogenoformans* | 67 |
| *Clostridium acetobutylicum* | 37 |
| *Clostridium perfringens* | 37 |
| *Clostridium tetani* | 37 |
| *Desulfitobacterium hafniense* | 38 |
| *Moorella thermoacetica* | 57 |
| *Thermoanaerobacter tengcongensis* | 75 |

Table S2 Archaeal genomes and Optimal Growth Temperature

| **Species** | **OGT** |
| --- | --- |
| *Methanocaldococcus jannaschii* | 85 |
| *Methanococcus maripaludis S2* | 35 |
| *Methanopyrus kandleri AV19* | 98 |
| *Methanospirillum hungatei* | 35 |
| *Pyrococcus abyssi* | 96 |
| *Pyrococcus furiosis* | 100 |
| *Pyrococcus horikoshii* | 98 |

Table S3 correlations with OGT in bacterial and archaeal clades containing thermophiles.

| **Amino acid** | **Correlation bacteria** | **Correlation archaea** |
| --- | --- | --- |
| A | 0.32 | 0.13 |
| C | -0.81** | -0.72 |
| D | -0.60* | -0.53 |
| E | 0.37 | 0.77* |
| F | 0.047 | 0.17 |
| G | 0.18 | -0.11 |
| H | 0.026 | -0.43 |
| I | -0.27 | -0.18 |
| K | -0.16 | 0.11 |
| L | 0.45 | 0.86** |
| M | -0.69** | -0.91*** |
| N | -0.53 | -0.47 |
| P | 0.50* | 0.48 |
| Q | -0.057 | -0.70 |
| R | 0.37 | 0.50* |
| S | -0.62** | -0.98*** |
| T | -0.58 | -0.96*** |
| V | 0.68* | 0.60 |
| W | 0.16 | 0.70 |
| Y | 0.025 | 0.35 |
| IVYWREL | 0.89*** | 0.94** |

* P < 0.1; ** P < 0.05; ***P < 0.005

# Supplementary figure legends

**Figure S1 Phylogenetic tree of Sordariomycetes.** A maximum likelihood tree was calculated with RaXML based on the concatenated alignments of 2,064 single copy orthologs in Sordariomycetes. Numbers on the branches indicate bootstrap support.

**Figure S2 Intergenic length distribution of *N. crassa*, *C.globosum* and *C.thermophilum****.* Intergenic regions of C. thermophilum (blue) are significantly smaller than Neurospora crassa (red) and Chaetomium globosum (green), due to genome compaction.

**Figure S3 Thermostability of Wild-type and Mutant ctArx1.** The critical temperature for thermostability is higher at lower protein concentration. The thermostability test (in vitro aggregation assay) with ctArx1 mutant proteins was performed at a 6-fold lower concentration (~1.3 mg/ml) than in **Fig. 4B**. ctArx1-nondestabilizing and ctArx1-destabilizing with five neutral or adaptive mutations, respectively (see **Figure 4A, B**), and ctArx1 wild-type recombinant proteins were affinity-purified and incubated at the indicated temperatures for 1 hour, separated into supernatant (S) and pellet (P) fractions by centrifugation and subjected to SDS-PAGE and Coomassie stain in comparison to the input (I). PS: protein standard


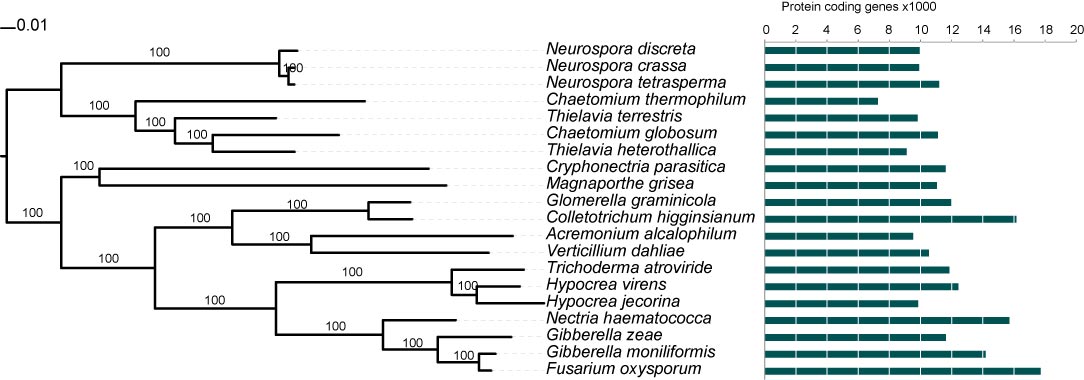


Figure S1


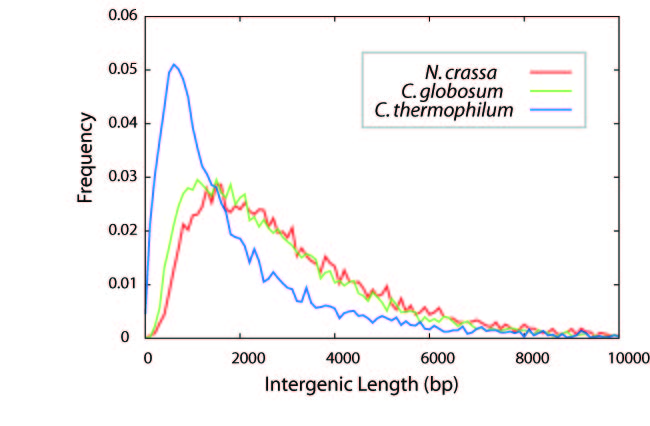


Figure S2


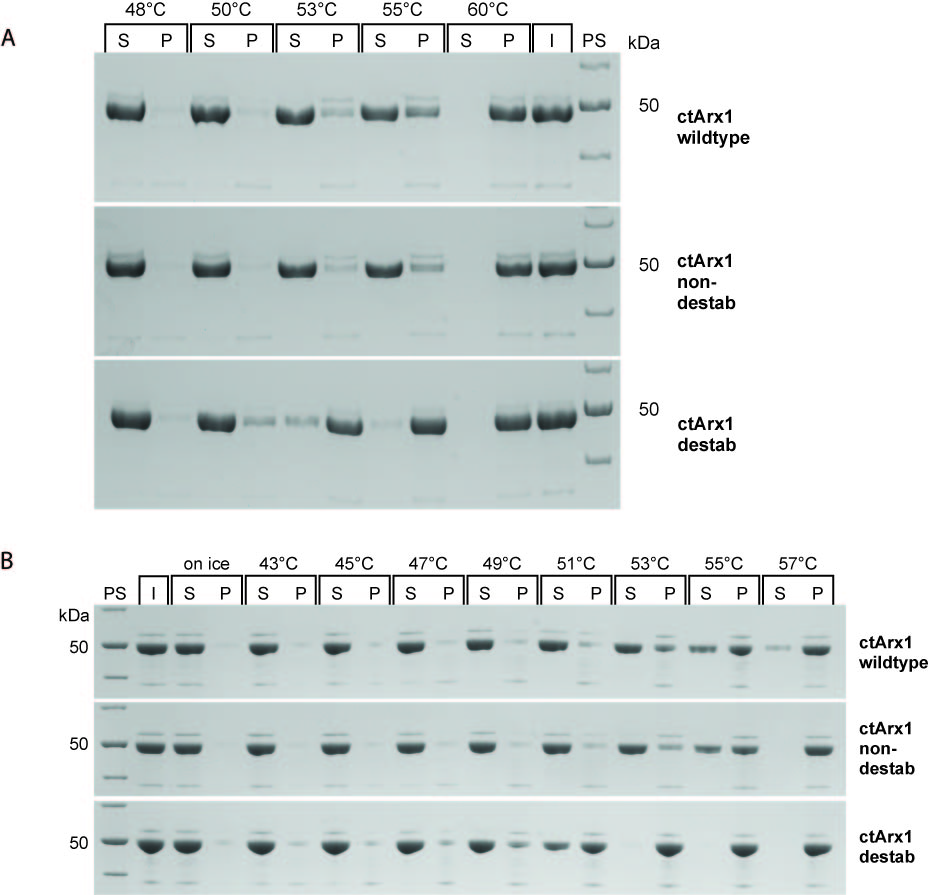


Figure S3
